# An improved genome reference for the African cichlid, *Metriaclima zebra*

**DOI:** 10.1186/s12864-015-1930-5

**Published:** 2015-09-22

**Authors:** Matthew A. Conte, Thomas D. Kocher

**Affiliations:** Department of Biology, University of Maryland, College Park, MD 20742 USA

**Keywords:** African cichlid fish, Genome assembly, Pacific Biosciences SMRT sequencing, Transposable elements

## Abstract

**Background:**

Problems associated with using draft genome assemblies are well documented and have become more pronounced with the use of short read data for *de novo* genome assembly. We set out to improve the draft genome assembly of the African cichlid fish, *Metriaclima zebra,* using a set of Pacific Biosciences SMRT sequencing reads corresponding to 16.5× coverage of the genome. Here we characterize the improvements that these long reads allowed us to make to the state-of-the-art draft genome previously assembled from short read data.

**Results:**

Our new assembly closed 68 % of the existing gaps and added 90.6Mbp of new non-gap sequence to the existing draft assembly of *M. zebra*. Comparison of the new assembly to the sequence of several bacterial artificial chromosome clones confirmed the accuracy of the new assembly. The closure of sequence gaps revealed thousands of new exons, allowing significant improvement in gene models. We corrected one known misassembly, and identified and fixed other likely misassemblies. 63.5 Mbp (70 %) of the new sequence was classified as repetitive and the new sequence allowed for the assembly of many more transposable elements.

**Conclusions:**

Our improvements to the *M. zebra* draft genome suggest that a reasonable investment in long reads could greatly improve many comparable vertebrate draft genome assemblies.

**Electronic supplementary material:**

The online version of this article (doi:10.1186/s12864-015-1930-5) contains supplementary material, which is available to authorized users.

## Background

Advances in high-throughput genome sequencing have allowed relatively inexpensive genome projects to be conducted for almost any organism. Projects such as the ‘Genome 10K Project’, which aims to sequence 10,000 vertebrate genomes [1], and the ‘Bird 10K’ project, which aims to sequence 10,500 bird species [2] have accelerated the production of draft genome sequences. Although attempts have been made to establish standards for declaring a genome sequence ‘complete’ [3], the quality of draft genomes varies dramatically. The limitations of using these draft genomes for downstream analyses have been documented [4, 5]. Still, it is clear that such draft genomes will continue to be the basis for genetic research on many species for the foreseeable future.

Short read sequencing technologies are appealing, as the cost per base is relatively cheap [6]. However, short reads (up to several hundred bp) make the *de novo* assembly process more difficult when the genome contains repeats that exceed the read length, which is typical for even relatively small genomes [7]. In addition, sequencing coverage biases caused by variation in base composition and PCR amplification further complicate the task of the assembler [8, 9]. Many different molecular biology and computational techniques have been developed that attempt to circumvent the problems associated with short read length, while keeping the cost of genome sequencing projects low. One technique is the use of paired-end and mate-pair jumping libraries. The power of this technique was demonstrated when a usable human draft genome assembly was produced using a combination of differently sized short read jumping libraries (180 bp to 40 kb) with the ALLPATHS-LG assembler [10].

The Assemblathon2 contest was organized as a friendly competition to assess current methods and evaluate the state of genome assembly by providing datasets of primarily short reads for three different vertebrate genomes. Assemblathon2 demonstrated that there was a lot of variability among submitted assemblies, and still plenty of room for improvement [11]. One of the three species used in the Assemblathon2 was the Lake Malawi cichlid fish, *Metriaclima zebra.* African cichlid fish are an ideal system for studying evolutionary mechanisms due to their phenotypic diversity and rapid speciation [12]. Draft genomes of *M. zebra* and four other African cichlid fish were recently published [13]. According to most assembly metrics, this *M. zebra* draft assembly (‘M_zebra_v0’) was among the best entries submitted to Assemblathon2. However, our extensive use of this assembly has revealed problems with gene models in or near assembly gaps, misassemblies encountered during the course of chromosome walks, and spurious spikes of differentiation statistics near gap and scaffold edges. These problems are not unique to this genome project, and complicate the use of many other draft genomes.

To improve the *M. zebra* draft assembly, we generated a 16.5× set of Pacific Biosciences SMRT (Single Molecule, Real-Time) sequencing reads. These ‘long’ PacBio reads can be used to improve draft assemblies by spanning gaps around repetitive regions and joining contigs and scaffolds [14]. Here we set out to improve the M_zebra_v0 genome assembly both to create a better reference assembly for the cichlid research community and to explore the improvements made possible with the addition of 16.5× of PacBio reads to even a relatively good draft vertebrate genome assembly.

## Methods

### Overview

**Fig. 1:**
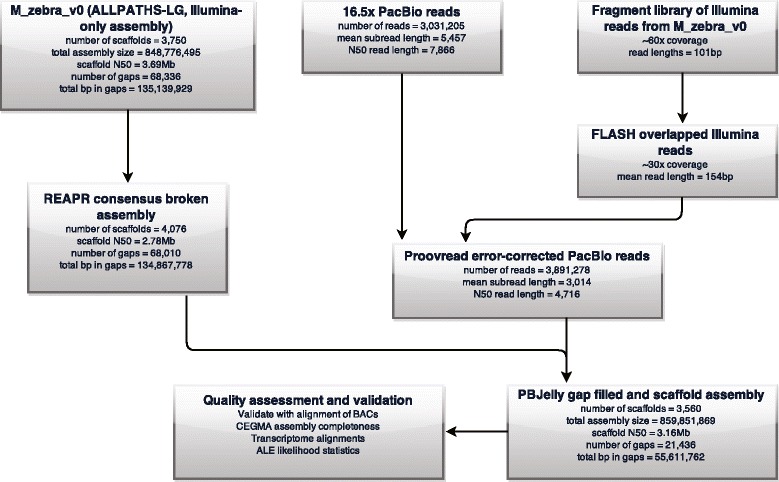
Genome assembly overview. Input datasets and the various steps involved in the assembly of M_zebra_UMD1 are diagrammed along with relevant metrics provided at each step

Our new ‘M_zebra_UMD1’ assembly is based on the recently published M_zebra_v0 assembly [13], made available by the Broad Institute [15]. We identified misassemblies in the M_zebra_v0 assembly as regions poorly supported by the existing Illumina mate-pair libraries. The assembly was ‘broken’ at these locations. A newly generated 16.5× coverage PacBio read set was error-corrected to improve base accuracy and identify potentially chimeric reads. These corrected PacBio reads were then used to fill in gaps and to join together scaffolds in the broken M_zebra_v0 assembly. The new M_zebra_UMD1 assembly was then evaluated by comparison to the sequence of individual bacterial artificial chromosome (BAC) clones, alignment of independently assembled transcriptomes, and assembly completeness and likelihood statistics. Figure 1 provides an overview of this assembly process with several assembly statistics shown at each step. Additional details of the steps in this process are provided below.

### Illumina datasets

The M_zebra_v0 assembly was originally created using seven different Illumina insert size libraries [13] as input to the ALLPATHS-LG assembler [10]. Table 1 provides details of each of the different Illumina libraries used.

**Table 1:** Illumina insert libraries used for the original M_zebra_v0 ALLPATHS-LG assembly and here for REAPR breaking

Type	Library size (bp)	# of reads	# of bp	Sequence coverage
Fragment	180 +/− 15	597,610,332	60,358,643,532	60×
2–3 kb jump	2,218 +/− 363	492,188,542	49,711,042,742	50×
2–3 kb jump	2,738 +/− 352	217,999,666	22,017,966,266	22×
5 kb jump	4,362 +/− 625	147,317,752	14,879,092,952	15×
7 kb jump	6,080 +/− 759	158,260,012	15,984,261,212	16×
9 kb jump	8,099 +/− 1,345	143,454,662	14,488,920,862	14×
11 kb jump	9,079 +/− 2,388	114,671,088	11,581,779,888	12×
40 kb jump	38,038 +/− 4,331	38,364,464	2,762,241,408	2.8×
Total		1,909,866,518	191,783,948,862	192×

### REAPR consensus breaking

**Fig. 2:**
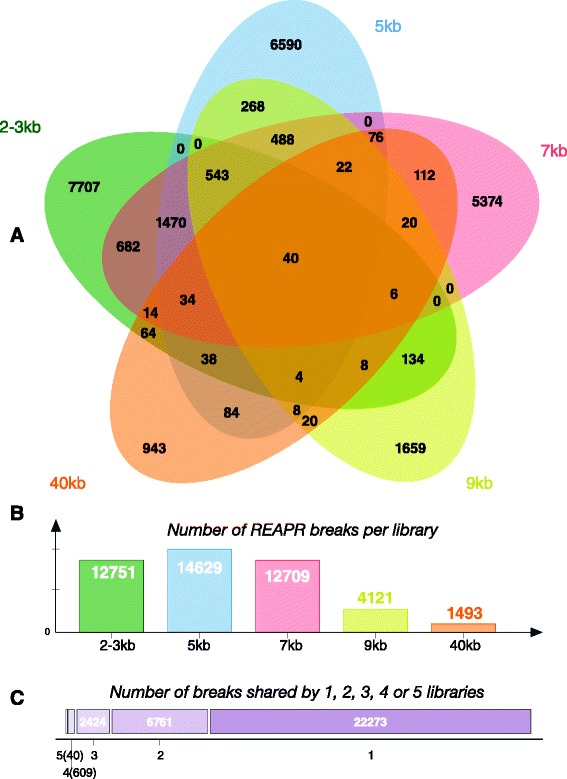
Overlap and number of REAPR breaks with different sized Illumina insert libraries. **a** Venn diagram showing the overlapping REAPR breaks generated by each of the different Illumina insert libraries provided in Table 1. **b** Histogram showing the total number of breaks for each library. The 11 kb Illumina library was omitted as it produced far more breaks (35,135) than the other libraries and was less complex overall. **c** Chart showing the number of REAPR breaks shared by a particular number of libraries (40 breaks shared by all 5 libraries, 609 breaks shared by exactly 4 libraries, 2424 breaks shared by exactly 3 libraries, etc.)

Recognizing Errors in Assemblies using Paired Reads (REAPR) is a tool that uses paired-read libraries to evaluate genome assembly accuracy, flag regions with potential errors, and break incorrectly joined scaffolds [16]. We ran REAPR version 1.0.17 on the M_zebra_v0 assembly using each of the libraries in Table 1 separately. First, the REAPR ‘*smaltmap’* task was run to align each of the libraries to the M_zebra_v0 assembly using SMALT version 0.7.6. The alignments for the two separate 2–3 kb libraries listed in Table 1 were merged using the ‘*samtools merge*’ command. The REAPR ‘*perfectfrombam*’ task was run on the SMALT alignment of the short-insert fragment library to generate read-depth information and identify repetitive regions. The REAPR ‘*pipeline*’ task was then run separately for each of the jump libraries. The high-quality short-insert alignment from the ‘*perfectfrombam*’ task was supplied to the ‘*pipeline*’ task for each of the jumping libraries. Aggressive breaking (‘*-break a=1’*) was also performed as it breaks scaffolds at regions where the fragment coverage distribution is low and potentially misassembled. The output of the REAPR ‘*pipeline*’ task includes the locations where REAPR broke the M_zebra_v0 assembly. Locations in the M_zebra_v0 assembly that were broken by a majority (four or more) of the insert libraries were compiled and the M_zebra_v0 assembly broken based on this consensus. A Venn diagram of the overlap of REAPR breaks between the libraries (Fig. 2) was created using jvenn [17].

In addition to breaking the M_zebra_v0 assembly using REAPR, we also randomly broke the assembly to evaluate how well random breaks could be put back together with the PacBio reads. The M_zebra_v0 assembly was randomly broken the same number of times as the REAPR-broken assembly described above.

### Pacific Biosciences SMRT sequencing

The Qiagen MagAttract HMW DNA kit was used to extract high-molecular weight DNA from a nucleated blood cell sample from a new individual from the same population used for the Broad Institute sequencing project. Size selection was performed at the University of Maryland Genomics Resource Center using a Blue Pippin pulse-field gel electrophoresis instrument. A library was constructed and 24 SMRT cells were sequenced on their PacBio RS II using the P5-C3 chemistry.

### Proovread error correction

Proovread is a hybrid error correction pipeline for correcting PacBio SMRT reads using short read data [18]. This step is important as the raw PacBio subreads are only ~85 % accurate [19] and contain chimeric reads at a rate of 1–2 % [20].

As shown in Fig. 1, we used the existing ~60× Illumina fragment library for Proovread error correction. This Illumina library was designed so that pairs would overlap and slightly longer reads could be generated. We first trimmed and filtered these reads using Trimmomatic version 0.32 with the following settings: *ILLUMINACLIP:TruSeq2-PE.fa:2:30:10 SLIDINGWINDOW:4:20 LEADING:10 TRAILING:10 CROP:101 HEADCROP:0 MINLEN:80*. The adaptor sequences used in the *TruSeq2-PE.fa* file are provided in Additional file 1. We then used FLASH [21] version 1.2.11 with a mismatch density of 0.15 (−*x 0.15*) to overlap the trimmed reads. These trimmed, filtered and overlapped Illumina reads were used for error correction with Proovread. Proovread version 2.10 was run with the following BWA mem ‘bwa-pre’ configuration settings: −*k 12 -W 20 -w 40 -r 1 -D 0 -y 20 -A 5 -B 11 -O 2,1 -E 4,3 -T 2.5 -L 30,30* and the following BWA mem ‘bwa-finish’ configuration settings: −*k 17 -W 18 -w 40 -r 1 -D 0 -y 20 -A 5 -B 11 -O 2,1 -E 4,3 -T 3.5 -L 30,30.*

### Gap closure and scaffolding with PBJelly

PBJelly is a pipeline for improving genome assemblies using PacBio reads [14]. PBJelly version 14.9.9 was run using the error corrected PacBio reads as described above. This set of error corrected reads also included a portion of the raw PacBio reads where there was no Illumina coverage and no error correction could be performed. This maximized the use of the PacBio reads, by using both the error corrected PacBio reads as much as possible, while still using the remaining portions that could not be corrected. The initial PBJelly ‘setup’ step was run with the ‘--minGap’ parameter set to 19 to reflect the smallest gap size in the M_zebra_v0 assembly. The PBJelly ‘mapping’ step aligned the corrected PacBio reads to the consensus REAPR broken M_zebra_v0 assembly using BLASR [22] version 1.3.1.127046 and the following parameters: −*minMatch 8 -minPctIdentity 70 -bestn 1 -nCandidates 20 -maxScore −500 –noSplitSubreads.* The PBJelly ‘assembly’ step was run with the ‘--maxWiggle' parameter set to 2000 to account for predicted gap size error in the M_zebra_v0 assembly. The other PBJelly steps (‘support’, ‘extraction’, ‘output’) were run with default parameters.

### Quality assessment and validation

GMAP [23] version 2014-12-06 was used to align existing RNA-seq transcriptome assemblies of eleven *M. zebra* tissues. The transcriptome assemblies were created using Trinity [24] as part of the cichlid genome project [13] and made available as supplementary information [25].

Three BAC clones that were previously sequenced and assembled using Sanger sequencing technology were aligned to the existing and newly produced assemblies for validation. These published BACs correspond to several opsin gene loci: SWS2A/SWS2B/LWS (GenBank accession JF262084.1, 107.6kbp), SWS1 (GenBank accession JF262085.1, 77.6kbp), and RH2B/RH2A (GenBank accession JF262089.1, 83.5kbp) [26]. The BAC sequences were aligned to the corresponding M_zebra_v0 and M_zebra_UMD1 assembly sequences using Gepard [27] version 1.30 to create dotplots for comparison.

Completeness of the intermediate and final M_zebra_UMD1 assemblies was assessed using CEGMA [28] version 2.5 optimized for vertebrate genomes (−−vrt). CEGMA relied on GeneWise version 2.4.1, HMMER version 3.1b1, and NCBI BLAST+ version 2.2.29+. The 248 mostly highly conserved core eukaryotic gene set provided by CEGMA was used.

The likelihoods of the intermediate and final M_zebra_UMD1 assemblies were evaluated using ALE [29]. Each of the Illumina libraries were aligned to the assemblies using Bowtie2 [30] version 2.0.2 with the ‘--very-sensitive’ preset parameter. The uncorrected PacBio reads were aligned to assemblies with BLASR version 1.3.1.127046 using the same parameters used above for PBJelly and the ‘-sam’ option to produce a SAM file for input to ALE. ALE was then run on each of the respective alignment files to produce likelihood and mapping statistics for each library.

Summary statistics of the assemblies were compiled using the assemblathon_stats.pl script [31].

### RepeatMasker comparisons

RepeatModeler [32] version open-1.0.8 was used to identify and classify *de novo* repeat families in each of the respective assemblies. To obtain a reasonable comparison, RepeatModeler was run using both the M_zebra_v0 and M_zebra_UMD1 assemblies separately. The consensus repeat sequences generated by RepeatModeler for each assembly were combined with the Repbase RepeatMasker library version 20140131. RepeatMasker [33] version open-4.0.5 was run with NCBI/RMBLAST version 2.2.27+ using the ‘-lib’ option to specify the respective RepeatModeler and Repbase combined library so that repeats predicted for M_zebra_v0 were modeled using the M_zebra_v0 assembly and repeats predicted for M_zebra_UMD1 were modeled using the M_zebra_UMD1 assembly.

## Results and discussion

### REAPR consensus breaking identifies misassemblies in M_zebra_v0

A genetic linkage map of *M. zebra* consisting of 834 RAD-tag markers was previously constructed [34]. Comparison of this map to the original M_zebra_v0 assembly identified a misassembly on the largest scaffold (scaffold_0). Table 2 shows the alignment of scaffold_0 to markers on two separate constructed linkage groups (LG7 and LG14) within the genetic map. Based on the map data we narrowed the location of the misassembly to a 1.7Mbp region between 3,426,502 (LG14) and 5,124,400 (LG7) on scaffold_0.

**Table 2:** Genetic markers that map to scaffold 0 of the M_zebra_v0 assembly

Marker name	Linkage Group	Map Position (cM)	Position on scaffold_0
33761	14	8.093	29,187
36558	14	7.385	169,879
12821	14	14.980	821,093
36086	14	9.480	937,855
47854	14	3.352	1,085,027
32200	14	2.455	1,988,503
55726	14	6.711	3,426,502
MZ371	7	64.131	5,124,400
Ed1012	7	58.564	13,037,865
UNH973	7	55.946	15,726,268

Markers on LG7 and LG14 are ordered by their position aligned to scaffold_0 of M_zebra_v0

Within this 1.7Mbp region there was a 19 bp gap at scaffold_0:3,622,144 where REAPR also predicted a misassembly for 5 out of the 6 Illumina insert libraries listed in Table 1. The 40 kb library was the only library where REAPR did not predict a misassembly. The 40 kb library was also the only jumping library that had mate-pairs that properly spanned this gap. REAPR predicted a misassembly at this gap for the other 5 jumping libraries either because they did not have spanning mate-pairs, had mate-pairs improperly oriented, and/or had mate-pairs aligning at a distance much different than the expected insert size. This small 19 bp gap also had no PacBio reads that spanned it. It is likely that this is the exact location of the misassembly identified by the genetic map data.

In addition to this known misassembly, REAPR identified many additional putative misassemblies in the M_zebra_v0 assembly. Figure 2 shows the number of breaks that REAPR predicted using the Illumina insert libraries listed in Table 1. Inspection of paired-read mappings from the 11 kb library revealed that it was much less complex than any of the other libraries. Using this 11 kb library, REAPR broke the M_zebra_v0 assembly 35,135 times. This was far more REAPR breaks than any other library and more than twice that of the 5 kb library (14,629 breaks). We elected to remove this 11 kb library from subsequent analyses.

The number of REAPR breaks shared by 5, 4 or more, 3 or more, 2 or more and 1 or more libraries was 40, 649, 3073, 9835 and 32107 respectively (Fig. 2). To begin our reassembly process we had to choose the appropriate number of REAPR breaks of the M_zebra_v0 assembly. Breaking the assembly too few times could leave unidentified misassemblies, while breaking too many times would fragment the assembly more than necessary. PacBio provides the SMRT View tool [35] for visualizing PacBio read alignments created using their BridgeMapper SMRT Pipe module within the SMRT-Analysis software suite [36]. The BridgeMapper module creates split read alignments with BLASR that can be used to identify misassemblies. Using these tools we were able to manually inspect the PacBio split read alignments and estimate that there are ~200-1000 misassemblies in the M_zebra_v0 assembly.

We also evaluated the rate of false positive breaks by comparing the number of REAPR breaks that could be re-joined with PBJelly and the corrected PacBio reads to the number of random breaks that could be re-joined with the same protocol. For the M_zebra_v0 assembly that was broken randomly, 541/649 (83.4 %) of the breaks were reassembled in the original M_zebra_v0 assembly order. In contrast, only 75 (11.6 %) of the 649 REAPR breaks were reassembled in the original M_zebra_v0 order. The random breaks are reassembled in the original order about 82 % of the time across all 5 libraries (Table 3). The percentage of REAPR breaks that are reassembled by PBJelly increases as the number of REAPR breaks increases, but is still far from the percentage of random breaks that were rejoined by PBJelly. It is clear that the consensus REAPR breaks have identified regions of the M_zebra_v0 assembly that were poorly supported and often misassembled. These regions are difficult to reassemble even with the corrected PacBio reads and likely represent complex and highly repetitive regions of the genome.

**Table 3:** REAPR and random breaks reassembled

Number of shared libraries	Number of breaks	REAPR breaks reassembled in M_zebra_v0 order	Random breaks reassembled in M_zebra_v0 order
5 out of 5	40	3 (7.5 %)	33 (82.5 %)
4 out of 5	649	75 (11.6 %)	541 (83.4 %)
3 out of 5	3,073	509 (16.6 %)	2,530 (82.3 %)
2 out of 5	9,835	2,135 (21.7 %)	8,024 (81.6 %)
1 out of 5	32,107	8,225 (25.6 %)	25,389 (79.1 %)

Based on the manual inspection of split read alignments and the rate of false positive breaks that were introduced we chose to break the M_zebra_v0 assembly wherever REAPR had predicted a misassembly in 4 or more of the Illumina insert libraries. This resulted in an assembly that was broken 649 times (40 breaks found in 5 or more libraries plus 609 breaks found in 4 or more libraries, Fig. 2).

### Proovread error correction

We generated a 16.5× set of PacBio reads using the P5-C3 chemistry. However, PacBio reads are error prone (80–85 % accuracy [9]) and known to contain chimeric reads at a rate higher than 1 % [20]. In addition, the SMRTbell adapter sequences are not always removed properly and may persist in up to 3 % of filtered PacBio reads depending on the sequencing protocol and library quality (Thomas Hackl, personal communication). These particular sequences are deemed “siameric” reads because they contain twin reads connected by the adapter. To detect and clip both chimeric and siameric reads, as well as improve the base-level accuracy of the PacBio reads, we ran Proovread [18]. The ~60× short-insert Illumina library was first overlapped to produce longer reads (mean overlapped read length = 154 bp, ~30× coverage) which were then used for the Proovread error-correction (Fig. 1). Additional file 2 provides summary statistics of the PacBio reads before and after the Proovread error-correction. While the mean and N50 read length decreased, Proovread detected raw PacBio reads that were potentially chimeric and siameric at the expected rates and split them at these junctions. This resulted in the number of raw reads increasing from 3,031,205 to 3,891,278 Proovread error-corrected reads. Any portion of raw PacBio reads that had no Illumina coverage were not split and were left in their original state. There was a tradeoff between having longer PacBio reads with a small percentage of chimeric reads or somewhat shorter but error-corrected PacBio reads. We chose to remove the chimeric reads and use the set of slightly shorter and error-corrected PacBio reads, especially considering the modest 16.5× coverage and the potential for chimeric/siameric introductions into the assembly in regions of low PacBio coverage.

### Gap filled assembly

Once the known and putative misassemblies were broken, and the errors in the PacBio reads were corrected, the M_zebra_v0 assembly was ready to be improved using PBJelly. Table 4 provides summary statistics of three assemblies: 1) the original M_zebra_v0 draft assembly, 2) M_zebra_v0 after being broken 649 times by REAPR, 3) and the broken assembly after gap-filling with PBJelly using the corrected PacBio reads (M_zebra_UMD1).

**Table 4:** Assembly summary statistics

Assembly	M_zebra_v0	REAPR broken	M_zebra_UMD1
Number of scaffolds	3,750	4,076 (+8.69 %)	3,560 (−5.07 %)
Total size of scaffolds	848,776,495	848,503,369 (−0.03 %)	859,851,869 (+1.3 %)
Longest scaffold	18,958,539	12,137,054 (−35.98 %)	14,997,410 (−20.89 %)
Mean scaffold size	226,340	208,171 (−8.03 %)	241,531 (+6.71 %)
N50 scaffold length	3,699,709	2,783,035 (−24.78 %)	3,158,421 (−14.63 %)
NG50^a^ scaffold length	3,007,690	2,252,862 (−25.10 %)	2,555,048 (−15.05 %)
Scaffold %N	15.93	15.9 (−0.19 %)	6.47 (−59.38 %)
Number of gaps	68,336	68,010 (−0.48 %)	21,436 (−68.63 %)
Non gap bp	713,636,566	713,635,591 (~0.00 %)	804,240,107 (+12.70 %)
Total gap bp	135,139,929	134,867,778 (−0.2 %)	55,611,762 (−58.85 %)
Number of exons mapped	4,490,849	4,490,529 (−0.01 %)	4,589,934 (+2.20 %)

^a^NG50 assumes genome size of 1.0Gb. Percentage change values in parenthesis are relative to M_zebra_v0

Most of the 649 REAPR breaks occurred at gaps. REAPR typically broke the M_zebra_v0 assembly twice, once on each side of the gap, generating 326 more scaffolds. This process effectively removed the gaps between these REAPR breaks. However, many of these broken scaffolds were put back together with the corrected PacBio reads in the new M_zebra_UMD1 assembly. The new assembly has 190 (5 %) fewer scaffolds relative to M_zebra_v0, and 516 (12.7 %) fewer scaffolds relative to the REAPR broken assembly. These may not seem like sizeable differences, but the M_zebra_v0 assembly was scaffolded using a ~40 kb jumping library, with a mean insert size (38,038 bp) that is longer than the longest error-corrected PacBio read in our dataset (33,000 bp). Therefore, since the M_zebra_v0 assembly was already relatively well placed into scaffolds, we did not see a large reduction in the number of scaffolds. We expect that draft assemblies that do not include mate pair libraries at this scale will experience a greater improvement in scaffolding using the long PacBio reads.

The total length of the M_zebra_UMD1 assembly increased by 11.1Mbp (+1.3 %) compared to M_zebra_v0. However, this leaves out the fact that 79.5 Mbp of gaps were filled, for a total of 90.6 Mbp of new sequence. The total length of the assembly contained in gaps decreased from 15.93 to 6.47 % of the assembly length, a 59 % improvement. The number of gaps decreased by 70 %, from 68,336 to 21,436. Further assembly metrics are provided in Additional file 3.

We mapped existing transcriptome assemblies from 11 tissues of *M. zebra* [13] to each of the genome assemblies using GMAP. The total number of mapped exons increased by 99,085 (+2.20 %, Table 4).

### Assembly completeness

**Fig. 3:**
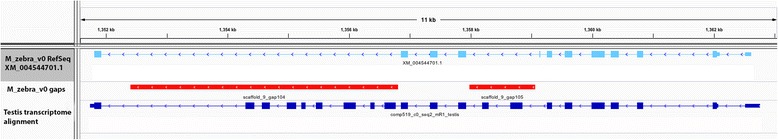
Gap filling improves gene models. The top (light-blue) track shows the original RefSeq gene model (XM_004544701.1) based on the M_zebra_v0 assembly aligned to the M_zebra_UMD1 assembly. The middle (red) track indicates the location of the gaps (now filled) in the original M_zebra_v0 assembly. The bottom (blue) track shows the testis transcriptome assembly aligned to M_zebra_UMD1 assembly and the additional 10 exons that were originally in gaps in the assembly

To assess the completeness of the assemblies we ran CEGMA [28], which scores the presence of 248 core eukaryotic genes (CEGs) in a given assembly. Table 5 provides the CEGMA completeness report for both the original M_zebra_v0 and the new M_zebra_UMD1 assemblies. The total number of complete plus partial CEGs is the same in both assemblies (237). However, the new M_zebra_UMD1 assembly contains 7 (2.6 %) more complete CEGs than the original M_zebra_v0 assembly. This increase in complete CEGs can be attributed to filling gaps that occur within gene models. One example of this was seen in the assembly of the predicted piwi-like protein (NCBI accession XM_004544701.1). Fig. 3 shows this piwi-like RefSeq mRNA sequence aligned to the M_zebra_UMD1 assembly. When the transcriptome assemblies were mapped to the M_zebra_UMD1 assembly, it became evident that the gaps in the original M_zebra_v0 assembly had left out at least 10 of the exons in the gene.

**Table 5:** Summary of CEGMA results

Assembly	M_zebra_v0	M_zebra_UMD1
Complete CEGs	227 (91.53 %)	233 (93.95 %)
% Of complete CEGs with multiple orthologs	25.55	26.61
Complete + Partial CEGs	237 (95.56 %)	237 (95.56 %)
% Of complete + partial CEGs with multiple orthologs	28.69	29.96
Total complete CEGs including putative orthologs	302	314
Average number of orthologs per complete CEG	1.33	1.35
Total complete + partial CEGs including putative orthologs	331	338
Average number of orthologs per complete + partial CEG	1.4	1.43

The new M_zebra_UMD1 assembly contains an increased number of CEGs that have multiple orthologs according to CEGMA (62, increased from 58). Some of these may represent paralogs that were collapsed in the M_zebra_v0 assembly and have been separately assembled in the M_zebra_UMD1 assembly. Extrapolated across the genome, the difference in the number of genes with multiple paralogs amounts to hundreds of new genes.

### Comparison with BACs from opsin loci

**Fig. 4:**
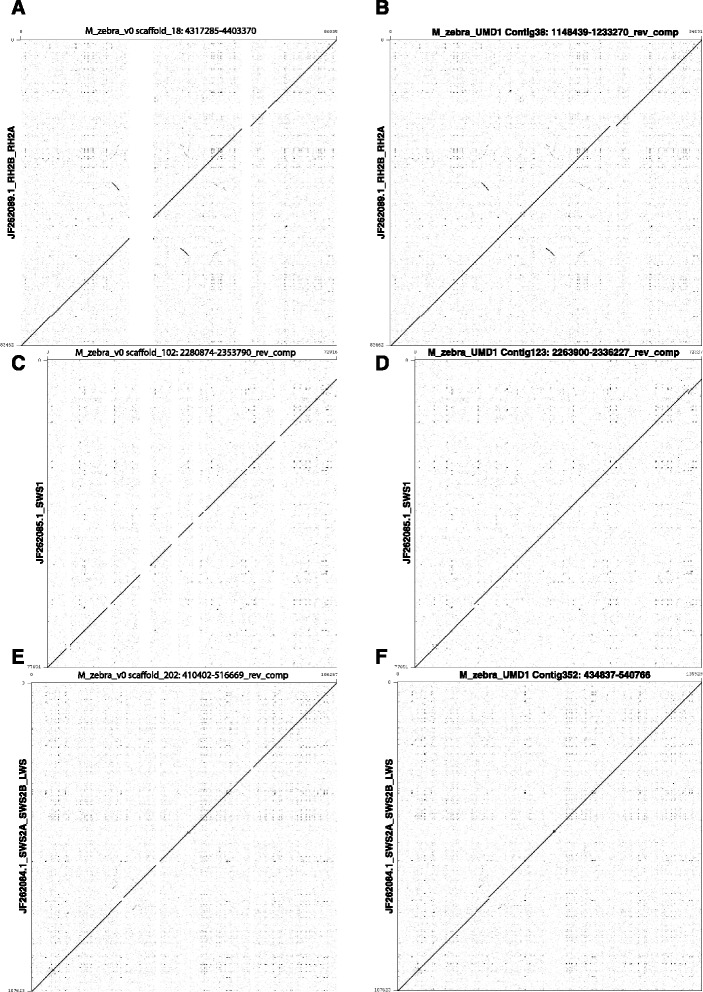
Dotplot alignments of opsin BACs to M_zebra_v0 and M_zebra_UMD1 to validate filled gap sequence. RH2B/RH2A (JF262089.1) versus M_zebra_v0 (**a**) and M_zebra_UMD1 (**b**). SWS1 (JF262085.1) versus M_zebra_v0 (**c**) and M_zebra_UMD1 (**d**). SWS2A/SWS2B/LWS (JF262084.1) versus M_zebra_v0 (**e**) and M_zebra_UMD1 (**f**)

Three *M. zebra* BAC clones previously sequenced and assembled using Sanger sequencing technology [26] were used to evaluate the accuracy of the error-correction and gap-filling procedures. Figure 4 shows dotplot alignments of these sequenced BACs to both the M_zebra_v0 and M_zebra_UMD1 assemblies. Most of the gaps in the M_zebra_v0 assembly have been filled in the M_zebra_UMD1 assembly. Several small gaps remain in the M_zebra_UMD1 assembly, as can be seen in Fig. 4b and d. BAC clone JF262085.1 (encompassing the SWS1 opsin) was the only BAC of the three that had gaps in the original assembled BAC sequence. The incongruence in the lower left portion of the Fig. 4d dotplot represents a difference in the size of the gap between the JF262085.1 BAC and the M_zebra_UMD1 assemblies. The abnormal alignment in the upper right portion of the dotplot in Fig. 4d represents a small 20 bp gap in the M_zebra_v0 assembly that has been “overfilled” by PBJelly with 779 bases. Both of these differences likely represent some structural sequence variation between the individual fish used for the BAC, M_zebra_v0 and M_zebra_UMD1 sequencing. These fish were collected from a natural population in Lake Malawi that has a small effective population size, so heterozygosity should be low, but some variation among individuals is expected.

### Assembly likelihood

The assembly summary metrics provided in Table 4 indicate the new M_zebra_UMD1 assembly is better in all respects except maximum scaffold length (−21 %) and scaffold N50 (−15 %). However, these decreases in continuity are accompanied by an overall improvement in accuracy and completeness of the assembly. To further quantify the accuracy of the new assembly we ran the Assembly Likelihood Evaluation (ALE) program [29]. This tool integrates read quality, mate-pair orientation, insert size, coverage and *k*-mer frequencies to provide a statistical measurement of assembly quality. Table 6 provides a summary of the ALE metrics calculated using several different read sets against both the M_zebra_v0 and M_zebra_UMD1 assemblies. The overall ALE likelihood score itself is not intended to be used to compare assemblies created from different datasets as is the case for the M_zebra_v0 (Illumina only) and M_zebra_UMD1 (Illumina + PacBio) assemblies. However, the remaining assembly metrics provided in the ALE output are very useful for comparison. For each Illumina library, the total number of placed reads is greater, the number of unmappable bases is lower, the number of unmappable regions is lower and the number of bases with 0 coverage is less in the M_zebra_UMD1 assembly compared to the M_zebra_v0 assembly. For brevity, only 3 of the 7 Illumina libraries are shown in Table 6, but the other Illumina libraries show the same trends (Additional file 4). A surprising amount of the genome had bases with 0 coverage alignment for the Illumina libraries. For example, the short-insert Illumina library had 121Mbp with 0 coverage (Table 6). Some of these regions with 0 coverage can be explained by the 55.6Mbp of gaps that remain in the M_zebra_UMD1 assembly, since ALE calculates gaps as bases with 0 coverage. The other ~66Mbp of non-gap sequence with 0 coverage (121Mbp minus 55.6Mbp for the short-insert Illumina library in Table 6) is mostly covered by the PacBio library. The PacBio library had about 10Mbp of non-gap sequence with 0 coverage and this reflects regions where the library either did not have any reads by chance or where only the Illumina libraries were able to sequence through. Additional PacBio coverage will help to more precisely describe such regions.

**Table 6:** Summary of assembly likelihood (ALE) results

	M_zebra_v0	M_zebra_UMD1
Illumina short insert library		
#Total Placed Reads	384,925,943	390,482,375
# Unmappable Bases	132,637,543	57,405,631
# Unmappable Regions	57,998	14,063
Bases with 0 Coverage	139,693,095	121,246,622
Illumina 2–3 kb insert library		
#Total Placed Reads	320,493,115	341,717,744
# Unmappable Bases	133,188,276	56,563,974
# Unmappable Regions	58,324	14,069
Bases with 0 Coverage	143,109,574	121,181,395
Illumina 40 kb insert library		
#Total Placed Reads	20,487,153	22,971,340
# Unmappable Bases	144,670,975	60,104,659
# Unmappable Regions	73,341	25,254
Bases with 0 Coverage	518,909,366	492,713,889
16.5× PacBio library		
#Total Placed Reads	2,703,712	2,794,402
Average Read Length (bp)	3,772	4,258
Average Read Overlap (bp)	3,453	3,886
# Unmappable Bases	82,472,941	45,023,176
# Unmappable Regions	18,363	6,349
Bases with 0 Coverage	114,035,849	65,141,623

### Analysis of transposable elements and repetitive sequences

**Fig. 5:**
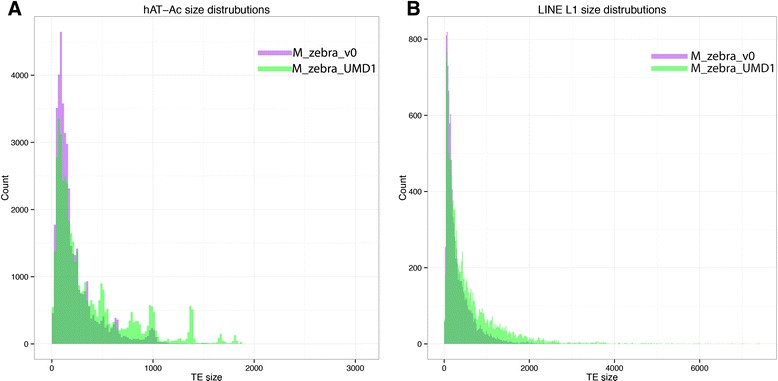
Gap filling improves both number and length of transposable element sequences. **a** Distribution of the size of DNA hAT-Ac transposable elements in the two assemblies. **b** Distribution of the size of the LINE L1 transposable elements in the two assemblies

A large amount of the sequence that was added in the new M_zebra_UMD1 assembly is composed of repetitive sequences and transposable elements that were either collapsed or not assembled in the original M_zebra_v0 assembly. We analyzed the total amount of repetitive sequences in both assemblies to understand the repeat content of the sequence that was added in M_zebra_UMD1. Table 7 lists several of the most abundant transposable element super families in the two assemblies. For most of the transposable element super families, the number of elements increased in the M_zebra_UMD1 assembly. Those transposable elements super families that decreased in number still increased in total bp, which means that the sequences of individual transposable element copies were longer in the M_zebra_UMD1 assembly. The assemblies of longer repeat copies can be seen for both the DNA hAT-Ac and LINE L1 transposable elements (Fig. 5). Additional file 5 provides a detailed list of hundreds of transposable elements and low complexity repeats that were annotated in both assemblies.

**Table 7:** Repetitive element summary

		M_zebra_v0				M_zebra_UMD1				Δ from M_zebra_v0			
order	super-family	number	bp	mean bp size	median bp size	number	bp	mean bp size	median bp size	number	bp	mean bp size	median bp size
DNA	TcMar-Tc1	133,563	30,394,950 (−3.58 %)	227.6	152	137,896	40,100,895 (4.66 %)	290.8	173	4,333	9,705,945	63.2	21
	hAT-Ac	41,018	9,251,093 (1.09 %)	225.5	143	43,310	16,553,134 (1.93 %)	382.2	215	2,292	7,302,041	156.7	72
LINE	L1	9,184	3,265,323 (0.38 %)	355.5	190	11,186	7,488,720 (0.87 %)	669.5	318.5	2,002	4,223,397	313.9	128.5
	L2	65,651	14,708,900 (1.73 %)	224.0	148	62,048	18,525,102 (2.15 %)	298.6	168	−3,603	3,816,202	74.5	20
	Rex-Babar	25,685	6,087,899 (0.72 %)	237.0	139	30,109	14,508,668 (1.69 %)	481.9	202	4,424	8,420,769	244.8	63
LTR	Gypsy	10,865	3,908,793 (0.46 %)	359.8	159	14,026	6,476,548 (0.75 %)	461.8	184	3,161	2,567,755	102.0	25
	Ngaro	3,955	393,178 (0.05 %)	99.4	94	10,633	1,841,475 (0.21 %)	173.2	157	6,678	1,448,297	73.8	63
SINE	MIR	12,756	1,741,837 (0.21 %)	136.6	111	10,900	2,395,459 (0.28 %)	219.8	165	−1,856	653,622	83.2	54
	tRNA-Core	7,419	953,921 (0.11 %)	128.6	124	12,054	1,819,302 (0.21 %)	150.9	145	4,635	865,381	22.4	21
Unknown		285,700	49,619,702 (5.85 %)	173.7	126	279,557	58,688,408 (6.83 %)	209.9	138	−6,143	9,068,706	36.3	12
Ancestral repeats		1,101,882	173,081,089 (20.39 %)			1,153,935	234,447,039 (27.27 %)				61,365,950		
Lineage specific		17,320	4,748,554 (0.56 %)			15,585	6,875,733 (0.80 %)				2,127,179		
Total		1,119,202	177,829,643 (20.95 %)			1,169,520	241,322,772 (28.07 %)				63,493,129

Compared to M_zebra_v0, the M_zebra_UMD1 assembly had fewer total lineage specific repeats identified (15,585 vs. 17,320), but a greater total amount of lineage specific repeat bases (6.9Mbp vs. 4.7Mbp). Again, this shows that longer lineage specific repeats have been assembled in the M_zebra_UMD1 assembly. In terms of total repetitive sequence, the new M_zebra_UMD1 assembly contained 63.5Mbp of additional sequence that was classified as repetitive. This is consistent with the idea that most of the gaps in the original M_zebra_v0 assembly spanned sequences consisting of transposable elements and other repetitive sequences.

## Conclusions

This study reports an improved assembly of the Lake Malawi African cichlid, *M. zebra*. We identified hundreds of misassemblies in the previous draft assembly [13]. We then used a newly generated set of 16.5× long PacBio reads to fill in 68 % of the previous assembly gaps and join together a portion of the previous scaffolds. This process added 90.6Mbp of new sequence to the assembly. Some of the newly added sequence contained gene sequence, allowing the identification of thousands of new exons. However, the majority of the newly added sequence was annotated as repetitive (70 %). The new data allowed us to assemble many more and longer copies of the transposable elements in the *M. zebra* genome. We hope this study can serve as an example of how a reasonable investment in long-read sequencing can improve even a relatively well-assembled vertebrate draft genome.

## Availability of supporting data

### Data availability

The *M. zebra* assemblies are available under NCBI BioProject ‘PRJNA60369’. The raw PacBio reads are available under the NCBI SRA accession SRX985423.

## Additional files

Additional file 1:
**Truseq2-PE.fa Illumina adapters used for Trimmomatic trimming.** (CSV 566 bytes) Additional file 2:
**PacBio read statistics before and after Proovread error-correction.** (XLSX 37 kb) Additional file 3:
**Extended assembly statistics.** (XLSX 10 kb) Additional file 4:
**Extended ALE scores.** (XLSX 50 kb) Additional file 5:
**Extended repeat results.** The repetitive elements included in Table 7 are highlighted in this file. (XLSX 54 kb)

## Abbreviations

BAC: Bacterial artificial chromosome
CEG: Core eukaryotic gene
cM: Centimorgan
PacBio: Pacific Biosciences
SMRT: Single Molecule, Real-Time
